# 

**Published:** 1868-04

**Authors:** 


### BIBLIOGRAPHICAL NOTICES.

*A Treatise on Odontalgia.*—By S. Parson Shaw. London, Trubner and Co. Manchester, Palmer and Howe. Under this title the author, who has evidently received his professional education in America, explains his object to be the presentation of a work so clear and precise, that the physician, surgeon and dentist may alike be able to detect the symptoms of this affection of the teeth. That he has succeeded in his object, as far as regards many English practitioners, there can be but little doubt, while in America the work would be better suited to the public among whom our patients are to be found.

The principles laid down are in the main correct, and the author deserves credit for his efforts "to emancipate a noble profession from error and charlatanerie" and in combatting the foolish notions which patients often derive from incompetent practitioners. A just tribute is paid to Prof. Chapin A. Harris, from whose works and that of Prof. Bond quotations are made. The subject is treated of under the heads of Anatomy and Formation of the teeth; The Trifacial nerve; Decay of the Teeth; Exposure of the Pulp; Odontitis; Alveolar Abscess; Sympathetic Tooth-ache; Diagnosis of Tooth-ache; Prevention and Cure of Tooth-ache; Disease in the Antrum; Neuralgia, Tic-douloreux, &c.

*The Dental Office and Laboratory.* The *Dental Quarterly* hitherto edited by Dr. A. Tees and F. N. Johnson and published by Johnson & Lund, Philadelphia, has ceased to exist after reaching the 4th No. of its IV. volume, and in its place appears a monthly Dental Newspaper of eight pages with the above title. This paper presents a very neat appearance and the first number contains several able and interesting Articles, besides miscellaneous matter interesting to the profession. The publishers have our best wishes for the success of their enterprise.

### EDITORIAL DEPARTMENT.

*The Harris Lectures.*—These Leetures, during the past session of the Baltimore College of Dental Surgery, have proven a success, and the *Twenty-Ninth Annual Circular* just published gives the names of the following eminent practitioners, who have kindly consented to assist the Faculty in this work of Dental Education during the next Session of the College, which commences on the 15th of October, 1868.

W. H. Morgan, M. D., D.D.S., Nashville, Tennessee; W. H. Atkinson, M.D., D.D.S., New York; W. W. H. Thackston, M.D., D.D.S., Farmville, Virginia; S. H. Williams, D.D.S., Baltimore, Maryland; John Allen, M.D., D.D.S., New York; B. F. Arrington, D.D.S., Wilmington, North Carolina; Thomas B. Gunning, M.D., D.D.S., New York; Edward N. Harris, D.D.S., Boston, Massachusetts; F. Y. Clarke, D.D.S., Savannah, Georgia; B. Wood, M.D.,D.D.S., Albany, New York.

Want of space prevents us from answering a number of Queries which have been sent to us, but we hope to be able to reply to our correspondents in the May number of the Journal.

